# Microbiota/Host Crosstalk Biomarkers: Regulatory Response of Human Intestinal Dendritic Cells Exposed to *Lactobacillus* Extracellular Encrypted Peptide

**DOI:** 10.1371/journal.pone.0036262

**Published:** 2012-05-14

**Authors:** David Bernardo, Borja Sánchez, Hafid O. Al-Hassi, Elizabeth R. Mann, María C. Urdaci, Stella C. Knight, Abelardo Margolles

**Affiliations:** 1 Antigen Presentation Research Group, Imperial College London, Harrow, United Kingdom; 2 Departmento de Microbiología y Bioquímica de Productos Lácteos, Instituto de Productos Lácteos de Asturias, Consejo Superior de Investigaciones Científicas, Villaviciosa, Spain; 3 Laboratoire de Microbiologie et Biochimie Appliquée, Ecole Nationale Supérieure des Sciences Agronomiques de Bordeaux, Gradignan, France; Charité, Campus Benjamin Franklin, Germany

## Abstract

The human gastrointestinal tract is exposed to a huge variety of microorganisms, either commensal or pathogenic; at this site, a balance between immunity and immune tolerance is required. Intestinal dendritic cells (DCs) control the mechanisms of immune response/tolerance in the gut. In this paper we have identified a peptide (STp) secreted by *Lactobacillus plantarum*, characterized by the abundance of serine and threonine residues within its sequence. STp is encoded in one of the main extracellular proteins produced by such species, which includes some probiotic strains, and lacks cleavage sites for the major intestinal proteases. When studied *in vitro*, STp expanded the ongoing production of regulatory IL-10 in human intestinal DCs from healthy controls. STp-primed DC induced an immunoregulatory cytokine profile and skin-homing profile on stimulated T-cells. Our data suggest that some of the molecular dialogue between intestinal bacteria and DCs may be mediated by immunomodulatory peptides, encoded in larger extracellular proteins, secreted by commensal bacteria. These peptides may be used for the development of nutraceutical products for patients with IBD. In addition, this kind of peptides seem to be absent in the gut of inflammatory bowel disease patients, suggesting a potential role as biomarker of gut homeostasis.

## Introduction

The lack of immunity against food antigens and the commensal microbiota is usually defined as immune tolerance [1]. Dendritic cells (DCs), the most potent antigen-presenting cells, are *commanders-in-chief* of the immune system, determining the nature and type of immune responses [2]. Intestinal DCs are central in controlling immune tolerance in the gastrointestinal tract [3]–[5]. DCs also imprint homing markers on T-cells that they stimulate e.g. gut DCs induce gut-homing markers on T-cells, localizing immune responses to specific tissues [6]. Regulatory cytokine IL-10 is essential in preventing inflammatory and autoimmune pathologies and is crucial for maintenance of intestinal homeostasis [7], [8].

The intestinal microbiota interacts with the local immune system promoting the mechanisms of intestinal homeostasis in health [9]–[11]. In certain disorders such as inflammatory bowel disease (IBD), this homeostasis is disrupted leading to a deregulated immune activity in the gut [12]. Harnessing the contribution of pro- and/or prebiotics to gut homeostasis has been proposed as alternative or complementary treatment for patients with IBD [13]. Direct exposure of DCs *in vitro* to different commensal bacteria has variable effects on DC phenotype and function often promoting anti-inflammatory activity [4], implying immunomodulation by commensal bacteria acts via DC *in vivo* [14]. However, the molecular mechanisms through which commensal bacteria interact with the human host and exert such immunomodulatory properties have remained elusive.

Extracellular proteins of bacteria are currently being characterised as potential mediators between commensal bacteria and the human host [15]. Such proteins could have relevant roles mediating interaction with the local microenvironment, including communication with other bacteria and the host immune system and even modulating the maintenance of the mucosal barrier [16]. We hypothesize therefore that the dialogue between intestinal bacteria and DC is partially mediated by the secretion of soluble bacterial compounds (including proteins).

To that end, we used a model of *Lactobacillus plantarum* and human DCs. *L. plantarum* is a lactic-acid-producing bacterium (LAB) with the largest genome [17] within the genus. This size provides the species with a high versatility to bind to different surfaces, and a great capacity for adaptation to diverse environmental conditions [18]. *L. plantarum* can be found in a wide array of fermented foods in different geographical regions [19] and, in addition, some *L. plantarum* strains, such as 299 v or WCFS1, confer benefits on human health, thus being considered as probiotics [20], [21]. Noteworthy, *L. plantarum* modulates the gene expression profile of the human intestine *in vivo,* promoting mechanisms of immune tolerance [22]. In addition, there is some evidence of *L. plantarum* is effective in methotrexate-induced colitis and in regulating the motility of the gastrointestinal tract. The amino acid sequences of some extracellular proteins secreted by *L. plantarum*, have been characterized, although their precise bioactivity has not been described [23]–[25]. Therefore we have chosen this species as a candidate to evaluate our hypothesis of a host-microbiota cross-talk, mediated through soluble factors. Our results confirmed that *L. plantarum* secreted bioactive proteins with the capacity to modulate the phenotype and function of human intestinal DC, confirming that the immune system/microbiota crosstalk may be also elicited through soluble factors.

## Materials and Methods

### Culture Conditions

*L. plantarum* BMCM12 strain was propagated on MRS agar (Becton Dickinson France SAS, Le Pont-De-Claix, France). Isolated colonies were used to inoculate 10 ml of MRS broth, which were used for total DNA extraction. Strains *Lc. lactis* NZ9000, *Lc. lactis* NZ9000-pNZ8110 (harbouring the empty plasmid pNZ8110), *Lc. lactis* D1, and *Lc. lactis* ST, were propagated on GM17 (BD). Five µg/ml chloramphenicol were added to the medium as selective agent when appropriated, and all the cultures were incubated in aerobiosis at 30°C.

### Cloning of the Sequence Coding for STp in *Lactococcus Lactis*

Total DNA of *L. plantarum* BMCM12 was extracted and purified from overnight cultures using the DNeasy Blood & Tissue Kit (Qiagen Iberia S.L, Madrid, Spain), following manufacturer instructions. The internal gene sequence coding for the serine/threonine rich domain of the protein D1 was amplified using primers STF and STHTR (Table S1), the latter including the genetic information for the addition of a histidine tag to the C-terminal domain of the recombinant protein.

Plasmid pNZ8110, containing the lactococcal Usp45 signal peptide, was extracted from *Lc. lactis* NZ9000-pNZ8110 strain using the QIAGEN Plasmid Midi Kit (Qiagen), following the manufacturer instructions. PCR products and plasmid pNZ8110 were digested with NaeI (Promega, Madison, WI), and the latter was further dephosphorylated using alkaline fosfatase (Promega). Digestion products were ligated using T4 DNA ligase (Promega) and then transformed into *Lc. lactis* NZ9000. The clone *Lc. lactis* ST, was selected for further studies using chloramphenicol as a selective marker. Sequencing of the resulting plasmid was carried out in order to ensure that undesirable mutations were not generated, and the DNA sequence of the gene was deposited in the GenBank under the accession number HQ262414. This strain produced a recombinant STp. It carried one extra glycine at the N-terminal of the mature protein after cleavage by sortase (coming from codon GGC originated in the reconstitution of the NaeI restriction site after ligation; 5′-GCCGGC-3′).

### Protein Manipulations and STp Purification

The production of STp was induced by adding 40 ng/ml nisin at cultures of strain *Lc. lactis* ST in exponential phase of growth, usually at an Abs_600_ of 0.3. STp was purified from *Lc. lactis* ST supernatants as follows. Five hundred µl of an overnight culture were used to inoculate 50 ml of fresh GM17 broth (1% v/v). After proper induction, cells were collected by centrifugation (10,000 g for 5 min at 4°C), and supernantants were filtered (0.22 µm). Five ml of 10× purification buffer, containing 500 mM NaH_2_PO_4_, 1.5 M NaCl, 100 mM imidazole, pH 8.0 were added to 45 ml of filtered supernatant and mixed. Five hundred ml of Ni-NTA agarose (Qiagen) was added to the mix, and gently stirred at 4°C for 2 h. The mixture was then loaded into a column (BioRad), being washed twice with 4 ml of a wash buffer containing 50 mM NaH_2_PO_4_, 300 mM NaCl, 20 mM imidazole, pH 8.0. ST fragment was eluted with 2 ml (four fractions of 500 µl) of elution buffer (50 mM NaH_2_PO_4_, 300 mM NaCl, 250 mM imidazole, pH 8.0), yielding usually a final concentration of 1 mg/ml.

Fractions were extensively dialysed against phosphate buffer saline (PBS) and the purified STp identified by N-terminal degradation, preformed in a Procise 494 Protein Sequencer (Applied Biosystems, Foster City, CA). Contaminating LPS was discarded in the purified peptide after *Limulus amebocyte* lysate protocol following manufacturer’s instructions (Kinetic-QCL™ assay, Lonza, Basel, Switzerland).

### Detection of STp in Human Intestinal Microenvironment

Polyclonal serum against the purified ST was generated in the Central Facilities of the University of Oviedo (Spain). Briefly, a rabbit was immunised five times, with an interval of 15 days between immunisations, with 500 µg of protein dissolved in 1 mL of PBS, and mixed with 1 mL of Freund’s incomplete Adjuvant. The rabbit was finally sacrificed by intracardiac puncture and blood was let to coagulate at 37°C for 4 h and subsequent overnight incubation at 4°C. Serum was separated by centrifugation (30 min, 2000 g), and used for purifying the IgG. Ammonium sulphate was added to a final concentration of 45% (w/v), and the mix was incubated overnight at 4°C. After centrifugation (1 h, 10000 g, 4°C), the pellet was resuspended in 30 mL of PBS. This was extensively dialyzed against PBS, and loaded in a ProteinA Sepharose 4 Fast Flow, previously equilibrated with 10 column volumes of PBS (50 mL). The column was washed with 6 column volumes of PBS, and five fractions of 5 mL were eluted with citric acid 100 mM pH 3.0. pH was corrected in each aliquot by adding 1 mL of 1 M Tris-HCl pH 9.0. Fractions were mixed, centrifuged in a Vivaspin 20 device (3000 g, molecular weight cut-off of 10 kDa) and washed with 20 mL of PBS. Protein concentration was estimated by measuring the A_280_ of the sample, aliquoted and stored at −80°C.

The IgG fraction was used for the detection of STp-containing proteins in human intestinal microenvironment. Briefly colonic biopsies were obtained at colonoscopy from 10 healthy controls, following informed consent after ethical approval from the Outer West London Research Ethics Committee (United Kingdom). These patients had macroscopically and histologically normal intestines, and had been referred with symptoms of rectal bleeding or change in bowel habit. Biopsy specimens were collected in ice-chilled complete medium and cultured within 1 hour in complete medium [Dutch modified RPMI 1640 (Sigma-Aldrich, Dorset, UK) containing 100 u/mL penicillin/streptomycin, 2 mM L-glutamine, 50 µg/mL gentamicine (Sigma-Aldrich) and 10% foetal calf serum (TCS cellworks, Buckingham, UK)] in 24 well culture dishes (1 biopsy/ml/well) for 24 hours (37°C, 5% CO_2_, high humidity). Negative control involved the culture and handling in parallel of complete medium alone. A second negative control involved the use of human skin samples, following informed consent, of two patients during abdominal closure after colorectal surgery on non-cancer, non-IBD patients who were not genetically pre-disposed to colorectal cancer. Skin samples were collected in complete medium. Incubation with Dispase II (Sigma-Aldrich, St. Louis, USA) was used to separate the epidermal and dermal layers which were subsequently cultured for 24 hours (37°C, 5% CO_2_, high humidity). Media were centrifuged in all cases (1500 rpm, t = 5′) and cell-free biopsy culture supernatants (SN) used to detect STp-containing proteins. Protein concentration of SN was measured using the bicinchonic acid (BCA) Protein Assay kit (Pierce, Rockford, IL, USA), and extracted in Laemmli buffer 5 min at 90°C. STp-containing proteins were detected by western-blotting. Briefly, 5 µg of protein extract from cultured biopsies were electro-transferred to a PVDF membrane for 30 min at a constant intensity of 50 V. STp-containing proteins where detected with the specific IgG fraction as a primary antibody (1∶1000), and with a anti rabbit IgG (HRP conjugated) as secondary antibody (1∶2000) (Sigma). Pre-immunization serum, as negative control, confirmed the specificity of the reaction since it did not detect any STp-containing protein. Purified STp fragment (1 µg) was also detected with the same procedure using a Penta·His™ HRP conjugate from Qiagen. The Pierce CN/DAB Substrate Kit (Pierce), including both chloronapthol and diaminobenzidine, was employed as colorimetric substrate for HRP in all cases.

### Dendritic Cells from Peripheral Blood

*Human peripheral blood* was collected from healthy volunteers with no known autoimmune or inflammatory diseases, allergies or malignancies, following informed consent. Peripheral blood mononuclear cells (PBMC) were obtained by centrifugation over Ficoll-Paque Plus (Amersham Biosciences, Chalfont St. Giles, UK). Human blood enriched DC were obtained following NycoPrep™ centrifugation of overnight cultured PBMC. This protocol has been characterised in detail in previous studies from our laboratory as a way to obtain fresh human blood enriched DC [14]. Obtained cells display morphological characteristics of DC (both at light and electron microscopy), express HLA-DR and are potent stimulators of naïve T-cells. DC were cultured for 24 hours (0.5 million cells/ml) in complete medium in the presence of STp (10 µg/ml, 1 µg/ml and 0.1 µg/ml) or LPS (100 ng/ml) (Sigma-Aldrich, St. Louis, USA). Results were compared with a paired culture in basal medium which acted as an internal control.

### Intestinal Dendritic Cells

*Colonic biopsies* were obtained at colonoscopy from extra 8 healthy controls as previously detailed. Freshly obtained colonic biopsies were collected in ice-chilled complete medium and processed within an hour. Biopsies were incubated with Hanks’s balances salt solution (HBSS) (Gibco BRL, Paisley, Scotland, UK) containing 1 mM dithiothreitol (DTT) (Sigma-Aldrich) for 20 minutes and then in 1 mM ethylenediamine tetraacetic acid (EDTA) solutions to remove the associated mucus/bacteria and epithelial layer respectively. Lamina propia mononuclear cells were obtained from biopsy tissue following a quick digestion in the presence of 1 mg/mL of collagenase D (Roche Diagnostics Ltd, Lewes, UK) in complete medium which does not affect neither the phenotype nor the function of DC [4]. Total lamina propria mononuclear cells were incubated for 24 hours with or without the addition of 1 µg/ml of STp and compared to a basal culture. DC from total lamina propria mononuclear cells were identified by flow cytometry as HLA-DR^+^CD3^−^CD14^−^CD16^−^CD19^−^CD34^−^ [4].

### Antibody Labelling

Table S2 shows the specificity, clone and fluorochrome of the monoclonal antibodies used. Cells were labelled in phosphate-buffered saline (PBS) containing 1 mM EDTA and 0.02% sodium azide (FACS buffer). Labelling was performed in ice and dark for 20′. Cells were washed twice in FACS buffer, fixed with 1% paraformaldehyde in 0.85% saline and stored at 4°C prior to acquisition on the flow cytometer within 48 hours. Appropriate isotype-matched control antibodies were purchased from the same manufacturers.

### Flow Cytometry and Data Analysis

Cells were acquired on a FACSCalibur cytometer (BD Biosciences) and analysed using WinList 5.0™ software (Verity, ME, US). The proportion of cells positive for a given marker was determined by reference to staining with an isotype-matched control. For single parameter analysis WinList was used to subtract the normal cumulative histogram for isotype control staining from a similar histogram of staining with the test antibody using the superenhanced D_max_ (SED) normalised subtraction.

### Intracellular Cytokine Staining

The intracellular cytokine production by non-stimulated DC was measured using the superenhanced D_max_ (SED) normalised subtraction to subtract the normal cumulative histogram for cytokine staining without added monensin from a similar histogram of staining with cytokine and added monensin for the last 4 hours of cell culture (3 µM, Sigma, UK). Cells were subsequently labelled for surface markers on ice as previously explained, fixed with Leucoperm A and permeabilized with Leucoperm B before adding antibodies for intracellular labelling. After incubation cells were washed in FACS buffer, fixed and acquired as previously reported.

This sensitive technique has been validated by our research group and allows us to assess the natural on-going cytokine production (without external PMA and/or ionomycine stimulus) of DC [4]. By that approach we do not quantify the intracellular content of a given cytokine. Instead we determine ongoing cytokine production in a time window of 4 hours (monensin incubation) irrespectively of the initial cytokine content.

**Figure 1:**
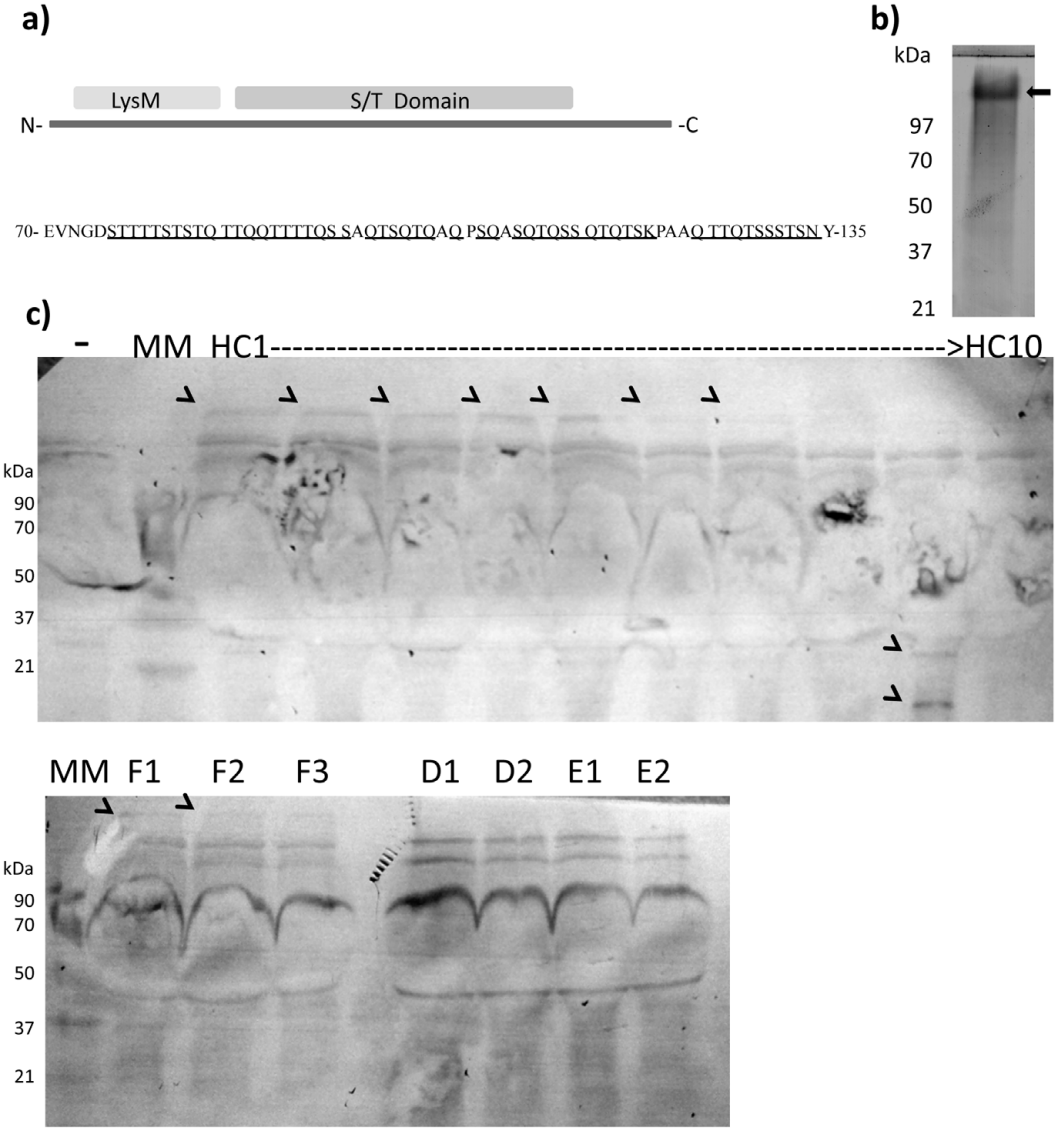
Structure, purification and location of the ST peptide. **a**) Domain structure of the protein D1, where the ST peptide is encoded (S/T domain); **b**) Western blot performed with a specific horseradish peroxidase-conjugated anti His_5_ antibody, showing the anomalous migration of the purified His-tagged ST peptide (marked with an arrow); **c**) Western blot using the polyclonal anti-STp serum as primary antibody; specific immunoreactive bands are labelled with arrows; -: complete medium (negative control), HC#: protein extracts obtained from culture supernatants of healthy colonic biopsies, F#: Some culture supernatants were 0.2 µm filtered prior to protein extraction, D# and E#: protein extracts from culture supernatants of human epidermal and dermal layer cultures respectively.

### Proliferation Assay

Freshly obtained PBMC from healthy controls were suspended in MiniMACs buffer (PBS containing 0.5% BSA and 2 mM EDTA). T-cells were enriched by depletion of CD14, CD19 and HLA-DR positive cells with immunomagnetic beads (Miltenyi Biotech, Bisley, UK) following manufacturer’s instructions. An average of 94.91%±1.06 (mean±SD) T-cells were obtained following enrichment. T-cells were labelled with 10 µM 5-carboxyfluorescein diacetate succinimidyl ester (CFSE, Invitrogen Ltd, UK) following manufacturer’s instructions. CFSE-labelled T-cells (4×10^5^/well) were incubated for 5 days in U-bottomed 96 well microtitre plates with enriched allogeneic gut or blood DC at 0%, 1%, 2% or 3%. Cells were recovered and CFSE^low^ proliferating cells identified, quantified and phenotyped by flow cytometry as described before [4].

## Results and Discussion

In the present work, we have characterized the interaction of a single peptide, encoded in the amino acid sequence of protein D1 (homologous to gi|28270057 from *L. plantarum* WCFS1) [25], with human DCs. The bioinformatic analysis of the D1 amino acid sequence revealed an internal region with a relatively high abundance of uncharged polar amino acids. This region had a predicted molecular mass of 6.8 kDa, and contained 22.7% of serine and 31.8% of threonine. Polar uncharged amino acids represented around 83% of the total amino acids (Figure 1a). Given the particular patterns of repeated serines and threonines, and its high content in these amino acids, we have denominated such region as STp and we produced and purified it using a recombinant *Lactococcus lactis* strain.

**Figure 2:**
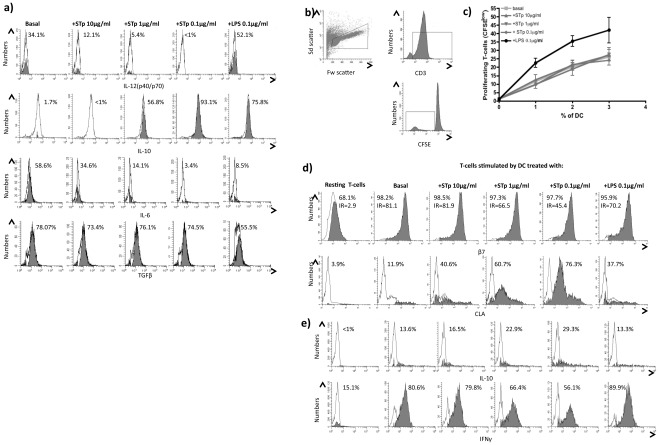
STp induced i) regulatory cytokines in blood enriched DC and ii) stimulated T-cells, which acquired a skin homing profile. **a**) Intracellular ongoing cytokine production of IL-12(p40/p70), IL-10, IL-6 and TGFβ in blood DC of healthy controls after 24 hours stimulation with STp (10 µg/ml, 1 µg/ml and 0.1 µg/ml) or LPS (100 ng/ml) compared to a basal culture. Closed histograms represent the percentage of positive cells assessed by intracellular cytokine staining and SED normalized subtraction from antibody stained cells cultured in the absence of monensin. That approach accurately quantifies the ongoing cytokine production of DC in a time window of 4 hours (monensin incubation) irrespectively of the initial cytokine amounts within the cells. **b**) Stimulatory capacity of DC was assayed in a mixed leukocyte reaction (MLR). T-cells were identified in the forward (Fw) and side (Sd scatter) and subsequent CD3 identification of dividing T-cells as CD3+ and CFSE^low^. **c**) Dose response proliferation of T-cells upon 5 days stimulation with different doses of allogeneic DC (0%, 1%, 2% and 3%) previously pulsed with different doses of STp or LPS compared to untreated DC (basal). Results show the mean±SEM of three independent experiments. **d**) Imprinted homing profile of stimulated T-cells (CFSE^low^) was determined regarding their surface expression and intensity ratio (IR) for the gut-homing integrin β7 and the skin-homing CLA molecules compared to resting T-cells cultured in the absence of DC. **e**) Acquired cytokine profile of such cells was determined as intracellular cytokine content of both IL-10 and IFNγ. Closed histograms represent the percentage of positive cells after subtraction from respective isotypes. All displayed histograms are representative of three independent experiments performed with similar results.

Serine-rich proteins from other microorganisms have been related to binding to eukaryotic components. For instance, the serine-rich fragment from the SrpA protein of *Staphylococcus aureus* mediates platelet-aggregation, although the predicted receptor is unknown [26]. Remarkably, the observed molecular mass of the purified STp in SDS-PAGE gels was over 100 kDa (Figure 1b). N-terminal sequencing by Edman degradation confirmed that this band corresponded to the STp. In certain cases, protein aggregates are difficult to disassociate, and this kind of artifacts may appear in SDS-PAGE, as happened with protein D1 [27].

STp was highly specific from *Lb. plantarum* species as revealed by BLASTP analysis against the cured non-redundant database of the NCBI (http://www.ncbi.nlm.nih.gov).

Further analysis of the amino acid sequence of the STp revealed that it did not contain cleavage recognition sequences for the major intestinal proteases (Figure S1). This was supported from the fact that neither pepsin nor trypsin digestions released any peptide, as revealed by tandem mass spectrometry (data not shown).

**Figure 3:**
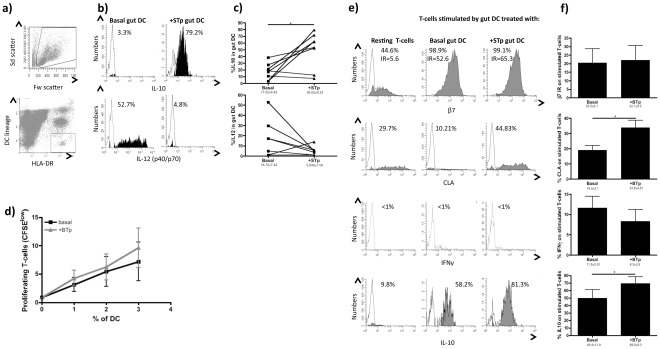
STp primes human intestinal DC towards a regulatory phenotype. **a**) DC were identified in total colonic lamina propria mononuclear cells from healthy controls by flow cytometry according to the Forward and Side scatters and the subsequent HLA-DR/lineage cocktail dot plot. DC were defined as HLA-DR^+^ and lineage^–^ (CD3, CD14, CD16, CD19 and CD34). **b**) Ongoing intracellular IL-10 and IL-12(p40/p70) cytokine production (closed histograms) was determined in colonic DC cultured with and without STp (10 µg/ml). Pooled data of 8 independent experiments are shown in panel **c**). **d**) Stimulatory capacity of such intestinal DC was determined upon 5 days culture in the presence of allogeneic CFSE-labelled T-cells as stated in Figure 2c. Results show the mean±SEM of 8 independent experiments. **e**) Imprinted homing profile (gut-homing: β7; skin-homing: CLA) and intracellular cytokine content (IL-10 and IFNγ) of stimulated T-cells (CFSE^low^) was compared to resting T-cells cultured in the absence of intestinal DC. Pooled data of eight independent experiments is shown in panel **f**). Closed histograms represent the percentage of positive cells after subtraction from respective isotypes. Lines and bars represent mean±SEM.

We thus investigated whether proteins containing the STp were produced by the commensal microbiota and therefore present in the human intestinal microenvironment. Colonic biopsies from healthy controls were cultured *in vitro* for 24 h in complete medium and the cell-free culture supernatant (SN) assayed with a polyclonal serum (IgG fraction) generated against the purified STp. Pre-immunization serum confirmed the specificity of the reaction since it did not detect any STp-containing protein. Western Blot analysis confirmed an immunoreactive high molecular mass band over 100 kDa in seven out of the ten cultures (Figure 1c) and two small molecular mass bands around 20 kDa in another healthy control. This high molecular band was not lost after 0.2 µm filtration of the SN (Figure 1d, samples F1→F3), and it was absent in SN from human epidermal and dermal layer cultures (Figure 1d, samples D# and E#). Our findings confirmed that protein containing regions homologous to STp can be found in the healthy human colonic microenvironment. Remarkably, STp-containing proteins were absent in the intestinal microenvironment from inflammatory bowel disease patients (unpublished data), suggesting a potential role as biomarker of gut homeostasis. This issue deserves further experimentation that is currently ongoing in our laboratories.

*In vitro* experiments confirmed that STp has the capacity to modulate phenotype and function of human DCs. Human blood DCs conditioned with STp acquired a regulatory cytokine profile (Figure 2a). Although STp conditioning did not alter the stimulatory capacity of DCs (Figure 2b and 2c), STp-pulsed DCs primed responding T-cells with a skin homing profile via induction of skin-homing molecule CLA (Figure 2d). T-cells stimulated by STp-pulsed DCs decreased the production of pro-inflammatory IFNγ and increased anti-inflammatory IL-10 production suggesting that these T-cells acquired an immunoregulatory profile (Figure 2e). All results were elicited in a dose dependent manner, with the greatest effects achieved at lower assayed doses (100 ng/ml) in all cases. Those effects were lost if the concentration was decreased further, and restored back to basal conditions (data not shown).

Having established that STp is a secreted peptide produced by *L. plantarum*, resistant to intestinal proteolysis, found in the human colonic microenvironment and capable of modulating phenotype and function of human blood-enriched DCs, we next studied its effect on human gut DCs. In contrast to effects on blood-enriched DCs, optimum effects of STp were elicited at a concentration of 1 µg/ml (data not shown). Similar to blood-enriched DCs, STp priming expanded ongoing production of regulatory IL-10 in human intestinal DCs (Figure 3a and 3b).

Since resting intestinal DCs from healthy controls do not usually produce pro-inflammatory cytokines like IL-12 [28], there was no statistically significant inhibition of such cytokine although its ongoing production was blocked in DCs from the 3 healthy controls producing it (Figure 3c). Human intestinal DCs are less stimulatory than blood DCs and prime T-cells with a gut-homing profile [29]. STp conditioning did not alter the stimulatory capacity of intestinal DC (Figure 3d). Nevertheless, such intestinal STp-pulsed DCs induced more CLA expression on stimulated T-cells than basal intestinal DC. IL-10 production by stimulated T-cells was also increased (Figure 3e–f). Recently, it has been proposed that the host has no capacity to distinguish between “harmful” and “commensal” microbiota, but the substrates that the microbiota produce actively promote immunologic tolerance to symbiotic bacteria [30]. Our data adds a new dimension to the concept of intestinal immune tolerance and shows that STp could be related not to the mechanisms of intestinal immune tolerance but rather of intestinal *immune ignorance* by diverting immune responses from the gastrointestinal compartment [10]. Therefore, in health, T-cells stimulated by bacteria-products-primed DC would be diverted away from intestinal sites to the skin.

Similar results highlighting the role of bacterial-derived products have been recently reported such as the role of immunomodulatory polysaccharide A from *Bacteroides fragilis* that mediated conversion of CD4+ T-cells into IL-10 producing T-cells [31], or the case of a soluble protein produced by *L. rhamnosus* GG, which prevented cytokine-induced apoptosis in intestinal epithelial cells [32]. Similarly, peptidoglycan of *Lactobacilli* was capable of inducing a regulatory phenotype on mouse intestinal DC [33] while probiotic bacterial DNA increases IL-10 production by DC, while DNA from non-probiotic bacteria failed to induce such regulatory phenotype on DC (Hart, AL; personal communication). Such evidence is in agreement with our findings and suggests that the crosstalk between the commensal microbiota and the local immune system is partially elicited through soluble factors and not exclusively through direct cell contact.

To sum up, in the human gut *L. plantarum* secretes an extracellular protein that releases an internal fragment (STp) when cleaved by intestinal proteases. STp might thus interact with human intestinal DCs *in vivo* promoting mechanisms of intestinal homeostasis. Further research using high-throughput techniques and *in vivo* experiments would shed light on the signaling pathways triggered by this peptide on the mucosal cells. Our ultimate aim is to characterise proteins, produced by probiotic bacteria, that are resistant to gut enzymes and produce ‘homeostatic’ effects on immunity. Such ‘natural’ products from commensal bacteria may well have been honed *in vivo* over millennia to facilitate mutually beneficial interactions between the microbiota and its host. In agreement with that concept we have identified a secreted bacterial peptide, highly resistant to proteolysis by gastrointestinal enzymes, which may play a role in generation of regulatory immune responses in the gut. From an applied point of view, STp may be used as an additive and/or nutraceutical compound and may therefore set the basis for non-drug related dietary treatment for patients with IBD. Its presence in the gut of healthy individuals, together with its absence in most of the IBD gut samples analysed so far, makes STp-containing proteins as promising biomarkers of healthy gut.

## Supporting Information

Figure S1 Slide 1: Theoretical cleavage sites of the intestinal proteases chymotrypsin, pepsin and trypsin were predicted at the ExPASy proteomic server, using the peptide cutter application (http://expasy.org/tools/peptidecutter/). The ST domain, where no predicted cleavage sites are predicted, is highlighted with the black arrow.
(PPT)

Table S1 Strains and primers used in the present work. * NaeI recognition sites are underlined, and sequence coding for the histidine tag double-underlined.
(DOC)

Table S2 antibodies used for flow cytometry.
(XLS)

## References

[1] Mowat AM (2003). Anatomical basis of tolerance and immunity to intestinal antigens.. Nat Rev Immunol.

[2] Banchereau J, Steinman RM (1998). Dendritic cells and the control of immunity.. Nature.

[3] Chirdo FG, Millington OR, Beacock-Sharp H, Mowat AM (2005). Immunomodulatory dendritic cells in intestinal lamina propria.. Eur J Immunol.

[4] Hart AL, Lammers K, Brigidi P, Vitali B, Rizzello F (2004). Modulation of human dendritic cell phenotype and function by probiotic bacteria.. Gut.

[5] Stagg AJ, Hart AL, Knight SC, Kamm MA (2003). The dendritic cell: its role in intestinal inflammation and relationship with gut bacteria.. Gut.

[6] Stagg AJ, Kamm MA, Knight SC (2002). Intestinal dendritic cells increase T cell expression of alpha 4 beta 7 integrin.. Eur J Immunol.

[7] O’Garra A, Barrat FJ, Castro AG, Vicari A, Hawrylowicz C (2008). Strategies for use of IL-10 or its antagonists in human disease.. Immunol Rev.

[8] Saraiva M, O’Garra A (2010). The regulation of IL-10 production by immune cells.. Nat Rev Immunol.

[9] Cerf-Bensussan N, Gaboriau-Routhiau V (2010). The immune system and the gut microbiota: friends or foes?.. Nat Rev Immunol.

[10] Feng T, Elson CO (2011). Adaptive immunity in the host-microbiota dialog.. Muc Immunol.

[11] Sansonetti PJ (2011). To be or not to be a pathogen: that is the mucosally relevant question.. Muc Immunol.

[12] Maloy KJ, Powrie F (2011). Intestinal homeostasis and its breakdown in inflammatory bowel disease.. Nature.

[13] Gourbeyre P, Denery S, Bodinier M (2011). Probiotics, prebiotics, and synbiotics: impact on the gut immune system and allergic reactions.. J Leukoc Biol.

[14] Ng SC, Plamondon S, Al-Hassi HO, English N, Gellatly N (2009). A novel population of human CD56+human leucocyte antigen D-related (HLA-DR plus) colonic lamina propria cells is associated with inflammation in ulcerative colitis.. Clin Exp Immunol.

[15] Lebeer S, Vanderleyden J, De Keersmaecker SCJ (2010). Host interactions of probiotic bacterial surface molecules: comparison with commensals and pathogens.. Nat Rev Microbiol.

[16] Sánchez B, Bressollier P, Urdaci MC (2008). Exported proteins in probiotic bacteria: adhesion to intestinal surfaces, host immunomodulation and molecular cross-talking with the host FEMS.. Immunol Med Mic.

[17] Chevallier B, Hubert JC, Kammerer B (1994). Determination of chromosome size and number of *rrn* loci in *Lactobacillus plantarum* by pulsed-field gel electrophoresis.. FEMS Microbiol Lett.

[18] Kleerebezem M, Boekhorst J, van Kranenburg R, Molenaar D, Kuipers OP (2003). Complete genome sequence of *Lactobacillus plantarum* WCFS1.. Proc Nat Acad Sci USA.

[19] Tallon R, Arias S, Bressollier P, Urdaci MC (2007). Strain- and matrix-dependent adhesion of *Lactobacillus plantarum* is mediated by proteinaceous bacterial compounds.. J Appl Microbiol.

[20] de Vries MC, Vaughan EE, Kleerebezem M, de Vos WM (2006). *Lactobacillus plantarum*: survival, functional and potential probiotic properties in the human intestinal tract.. Int Dairy J.

[21] Molin G (2001). Probiotics in foods not containing milk or milk constituents, with special reference to *Lactobacillus plantarum* 299 v.. Am J Clin Nutr.

[22] van Baarlen P, Troost FJ, van Hemert S, van der Meer C, de Vos WM (2009). Differential NF-kappa B pathways induction by *Lactobacillus plantarum* in the duodenum of healthy humans correlating with immune tolerance.. Proc Nat Acad Sci U S A.

[23] Beck HC, Madsen SM, Glenting J, Petersen J, Israelsen H (2009). Proteomic analysis of cell surface-associated proteins from probiotic *Lactobacillus plantarum*.. FEMS Microbiol Lett.

[24] Madsen SM, Glenting J, Vrang A, Ravn P, Riemann HK (2005). Cell surface-associated glycolytic enzymes from *Lactobacillus plantarum* 299v mediate adhesion to human epithelial cells and extracellular matrix proteins.. J Biotechnol.

[25] Sánchez B, Schmitter JM, Urdaci MC (2009). Identification of novel proteins secreted by *Lactobacillus plantarum* that bind to mucin and fibronectin.. J Mol Microbiol Biotechnol.

[26] Siboo IR, Chambers HF, Sullam PM (2005). Role of SraP, a serine-rich surface protein of *Staphylococcus aureus*, in binding to human platelets.. Infect Immun.

[27] Kankainen M, Paulin L, Tynkkynen S, von Ossowski I Reunanen J (2009). Comparative genomic analysis of *Lactobacillus rhamnosus* GG reveals pili containing a human-mucus binding protein.. Proc Nat Acad Sci U S A.

[28] Hart AL, Al-Hassi HO, Rigby RJ, Bell SJ, Emmanuel AV (2005). Characteristics of intestinal dendritic cells in inflammatory bowel diseases.. Gastroenterology.

[29] Mann ER, Bernardo D, Al-Hassi HO, English NR, Clark SK (2011). Human gut-specific homeostatic dendritic cells are generated from blood precursors by the gut microenvironment Inflamm Bowel Dis.

[30] Round JL, Lee SM, Li J, Tran G, Jabri B (2011). The toll-like receptor 2 pathway establishes colonization by a commensal of the human microbiota.. Science.

[31] Round JL, Mazmanian SK (2010). Inducible Foxp(3+) regulatory T-cell development by a commensal bacterium of the intestinal microbiota.. Proc Nat Acad Sci USA.

[32] Yan F, Cao H, Cover TL, Washington MK, Shi Y (2011). Colon-specific delivery of a probiotic-derived soluble protein ameliorates intestinal inflammation in mice through an EGFR-dependent mechanism.. J Clin Invest.

[33] Macho Fernandez E, Valenti V, Rockel C, Hermann C, Pot B (2011). Anti-inflammatory capacity of selected lactobacilli in experimental colitis is driven by NOD2-mediated recognition of a specific peptidoglycan-derived muropeptide.. Gut.

