# Supplementary material for: Microbiota/Host Crosstalk Biomarkers: Regulatory Response of Human Intestinal Dendritic Cells Exposed to Lactobacillus Extracellular Encrypted Peptide
Source: PLoS One. 2012 May 14;7(5):e36262. doi: 10.1371/journal.pone.0036262 (PMC3351486; doi:10.1371/journal.pone.0036262)
Supplement: Figure S1 — Slide 1: Theoretical cleavage sites of the intestinal proteases chymotrypsin, pepsin and trypsin were predicted at the ExPASy proteomic server, using the peptide cutter application (http://expasy.org/tools/peptidecutter/). The ST domain, where no predicted cleavage sites are predicted, is highlighted with the black arrow. (PPT) [file pone.0036262.s001.ppt]

## Slide 1
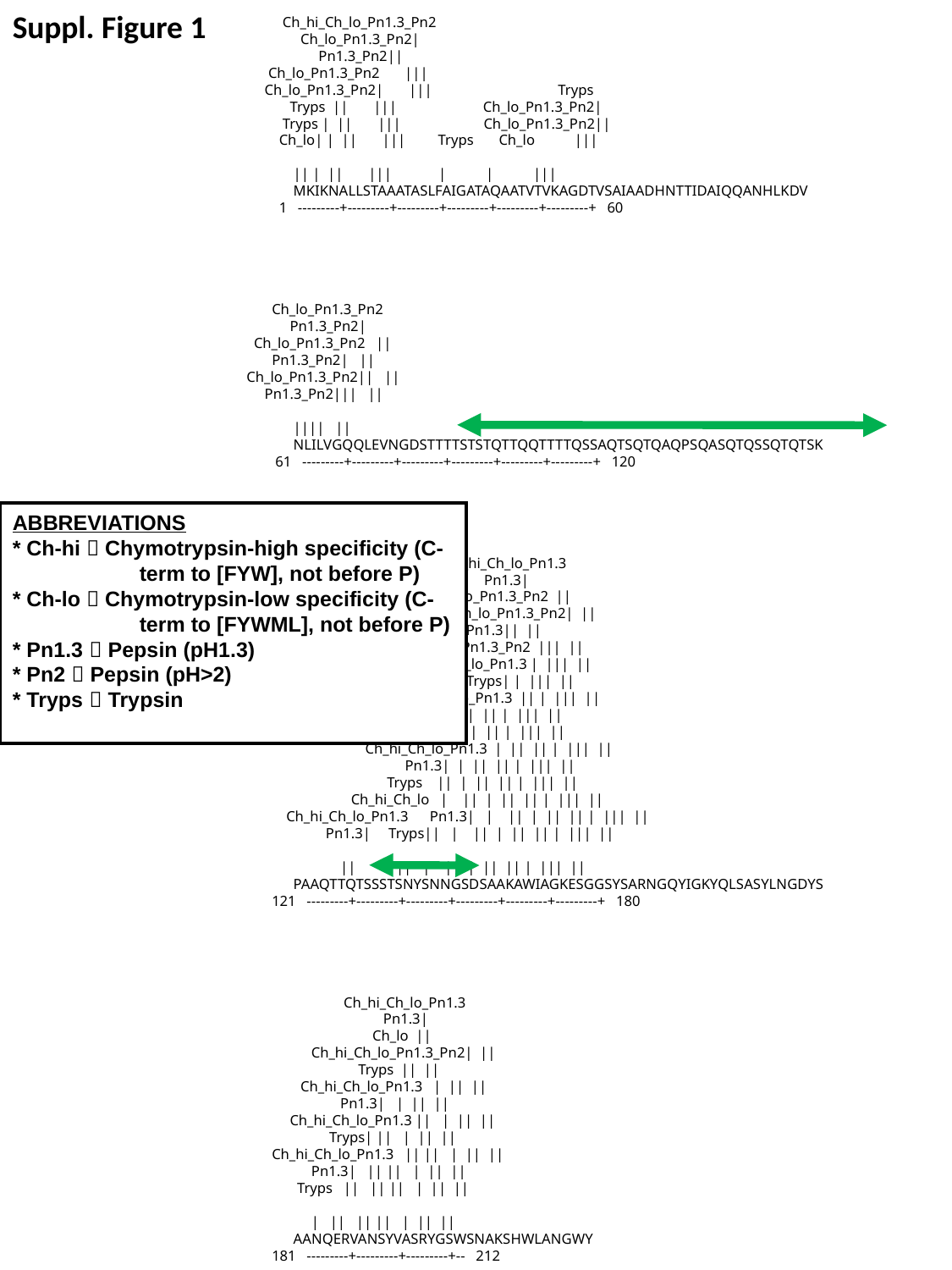

Suppl. Figure 1
 Ch_hi_Ch_lo_Pn1.3_Pn2
 Ch_lo_Pn1.3_Pn2|
 Pn1.3_Pn2||
 Ch_lo_Pn1.3_Pn2 |||
 Ch_lo_Pn1.3_Pn2| ||| Tryps
 Tryps || ||| Ch_lo_Pn1.3_Pn2|
 Tryps | || ||| Ch_lo_Pn1.3_Pn2||
 Ch_lo| | || ||| Tryps Ch_lo |||
 || | || ||| | | |||
 MKIKNALLSTAAATASLFAIGATAQAATVTVKAGDTVSAIAADHNTTIDAIQQANHLKDV
 1 ---------+---------+---------+---------+---------+---------+ 60
 Ch_lo_Pn1.3_Pn2
 Pn1.3_Pn2|
 Ch_lo_Pn1.3_Pn2 ||
 Pn1.3_Pn2| ||
 Ch_lo_Pn1.3_Pn2|| ||
 Pn1.3_Pn2||| ||
 |||| ||
 NLILVGQQLEVNGDSTTTTSTSTQTTQQTTTTQSSAQTSQTQAQPSQASQTQSSQTQTSK
 61 ---------+---------+---------+---------+---------+---------+ 120
 Ch_hi_Ch_lo_Pn1.3
 Pn1.3|
 Ch_lo_Pn1.3_Pn2 ||
 Ch_hi_Ch_lo_Pn1.3_Pn2| ||
 Pn1.3|| ||
 Ch_lo_Pn1.3_Pn2 ||| ||
 Ch_hi_Ch_lo_Pn1.3 | ||| ||
 Pn1.3_Tryps| | ||| ||
 Ch_hi_Ch_lo_Pn1.3 || | ||| ||
 Pn1.3| || | ||| ||
 Tryps || || | ||| ||
 Ch_hi_Ch_lo_Pn1.3 | || || | ||| ||
 Pn1.3| | || || | ||| ||
 Tryps || | || || | ||| ||
 Ch_hi_Ch_lo | || | || || | ||| ||
 Ch_hi_Ch_lo_Pn1.3 Pn1.3| | || | || || | ||| ||
 Pn1.3| Tryps|| | || | || || | ||| ||
 || ||| | || | || || | ||| ||
 PAAQTTQTSSSTSNYSNNGSDSAAKAWIAGKESGGSYSARNGQYIGKYQLSASYLNGDYS
 121 ---------+---------+---------+---------+---------+---------+ 180
 Ch_hi_Ch_lo_Pn1.3
 Pn1.3|
 Ch_lo ||
 Ch_hi_Ch_lo_Pn1.3_Pn2| ||
 Tryps || ||
 Ch_hi_Ch_lo_Pn1.3 | || ||
 Pn1.3| | || ||
 Ch_hi_Ch_lo_Pn1.3 || | || ||
 Tryps| || | || ||
 Ch_hi_Ch_lo_Pn1.3 || || | || ||
 Pn1.3| || || | || ||
 Tryps || || || | || ||
 | || || || | || ||
 AANQERVANSYVASRYGSWSNAKSHWLANGWY
 181 ---------+---------+---------+-- 212
ABBREVIATIONS
* Ch-hi  Chymotrypsin-high specificity (C-	term to [FYW], not before P)
* Ch-lo  Chymotrypsin-low specificity (C-	term to [FYWML], not before P)
* Pn1.3  Pepsin (pH1.3)
* Pn2  Pepsin (pH>2)
* Tryps  Trypsin
