# Supplementary material for: Microbiota/Host Crosstalk Biomarkers: Regulatory Response of Human Intestinal Dendritic Cells Exposed to Lactobacillus Extracellular Encrypted Peptide
Source: PLoS One. 2012 May 14;7(5):e36262. doi: 10.1371/journal.pone.0036262 (PMC3351486; doi:10.1371/journal.pone.0036262)
Supplement: Table S1 — Strains and primers used in the present work. * NaeI recognition sites are underlined, and sequence coding for the histidine tag double-underlined. (DOC) [file pone.0036262.s002.doc]

Supplementary table 1. Strains and primers used in the present work. * NaeI recognition sites are shown underlined, and sequence coding for the histidine tag double-underlined. Lb. *Lactobacillus*; Lc. *Lactococcus*.

| **Strains, primers and vectors** | **Features/sequence** | **Reference** |
| --- | --- | --- |
| *Lb. plantarum* BMCM12 | Natural D1 producer | 1 |
| *Lc. lactis* NZ9000 | Strain MG1363 with *nisRK* genes integrated in the chromosome. Host for plasmid pNZ8110-derivatives | 2 |
| *Lc. lactis* ST | Strain producing the ST-rich domain encoded in the *d1* gene from *Lb. plantarum* BMCM12. Cloned into pNZ8110. Chloramphenicol resistant. | This study |
| pNZ8110 | Vector for protein secretion using the signal sequence of protein Usp45 of *Lc. lactis*. Chloramphenicol resistant | 3 |
|  |  |  |
| STF* | GGGGCGCCGGCGAAGTAAATGGTGATAGCACT | This study |
| STHTR | GGGGCGCCGGCCTAGTGATGGTGATGGTGATGATAGTTTGATGTTGAACT | This study |
|  |  |  |

1. Sanchez, B., Schmitter, J.M. & Urdaci, M.C. Identification of Novel Proteins Secreted by Lactobacillus plantarum That Bind to Mucin and Fibronectin. *Journal of Molecular Microbiology and Biotechnology* **17**, 158-162 (2009).

2. Kuipers, O.P., de Ruyter, P.G.G.A., Kleerebezem, M. & de Vos, W.M. Quorum sensing-controlled gene expression in lactic acid bacteria. *Journal of Biotechnology* **64**, 15-21 (1998).

3. Mierau, I. & Kleerebezem, M. 10 years of the nisin-controlled gene expression system (NICE) in Lactococcus lactis. *Applied Microbiology and Biotechnology* **68**, 705-717 (2005).
